# Doing Feminist Research on Conflict, Violence and Peace: Ethical and Methodological Dilemmas

**DOI:** 10.1177/03058298241298689

**Published:** 2024-12-10

**Authors:** María Martín de Almagro, Julia Margaret Zulver, Priscyll Anctil Avoine, Nancy R. Tapias Torrado, Marie Berry

**Affiliations:** University of Ghent, Belgium; Swedish Defence University, Sweden; United College at the University of Waterloo, Canada; University of Denver, USA

**Keywords:** feminist methods, peace and conflict studies, ethical dilemmas, methodological dilemmas, dissonance, knowledge production, méthodes féministes, dilemmes éthiques et méthodologiques, production de la connaissance, metodologías feministas, dilemas éticos y metodológicos, producción del conocimiento

## Abstract

This piece offers a space for critical debate and reflection on the methodological and epistemological foundations that underpin feminist research on conflict, violence and peace. Taking stock of the variety of approaches and theoretical standpoints, we examine the (feminist) politics of knowledge production in academia and its limitations. We discuss how ontological and epistemological assumptions shape what counts as (feminist) academic knowledge and what is considered to be possible in (policy) practice. The article makes three contributions. First, we argue that the production of knowledge within disciplinary boundaries, and in particular, International Relations, is closely related to the discipline’s history of positivism and exclusion. Second, to counter that, we propose a close engagement with Black and decolonial feminist methods of feeling-knowing, storytelling and collaboration. Third, we highlight that embracing uncertainty means accepting incommensurability and heterogeneity, as well as a shift away from the urge to accumulate knowledge towards paying attention to the process of co-constructing it.

## Résumé

Cet article offre un espace de débat et de réflexion critiques sur les fondements méthodologiques et épistémologiques qui sous-tendent la recherche féministe sur les conflits, la violence et la paix. En tenant compte de la diversité des approches et des points de vue théoriques, nous examinons les politiques (féministes) de la production de connaissances dans le monde universitaire et ses limites. Nous abordons la manière dont les hypothèses ontologiques et épistémologiques façonnent ce qui est perçu comme des connaissances universitaires (féministes) et ce qui est considéré comme possible dans la pratique (politique). Cet article apporte trois contributions. Premièrement, nous soutenons que la production de connaissances au sein des limites disciplinaires, et en particulier au sein des RI, est étroitement liée à l’histoire positiviste et exclusive de la discipline. Deuxièmement, pour pallier cela, nous proposons un engagement étroit avec les méthodes féministes noire et décoloniale de production de connaissances par les émotions, la narration et la collaboration. Troisièmement, nous soulignons que le fait d’accueillir l’incertitude signifie accepter l’incommensurabilité et l’hétérogénéité du savoir, ainsi que renoncer au désir d’accumuler des connaissances pour prêter attention au processus de co-construction de celles-ci.

## Resumen

Este trabajo abre un espacio para el debate crítico y la reflexión sobre los fundamentos metodológicos y epistemológicos que sostienen la investigación feminista en torno al conflicto, la violencia y la paz. Hacemos un balance de la variedad de enfoques y posturas teóricas con el objetivo de examinar las políticas (feministas) de producción de conocimiento en el mundo académico y sus limitaciones. Examinamos cómo las presunciones ontológicas y epistemológicas definen qué cuenta como conocimiento académico (feminista) y qué se considera posible en la práctica (política). Este artículo ofrece tres aportaciones. En primer lugar, sostenemos que la producción de conocimiento dentro de los límites disciplinarios de las RR. II. está estrechamente relacionada con la historia de positivismo y exclusión de la disciplina. En segundo lugar, como contrapunto, proponemos una estrecha implicación con las metodologías feministas negra y decolonial del senti-pensar, la narración de historias y la colaboración. Por último, destacamos que aceptar y acoger la incertidumbre en el proceso de investigación significa admitir la inconmensurabilidad y la heterogeneidad del conocimiento, así como privilegiar su proceso de construcción colectiva, por encima de su acumulación.

## Introduction

This article is the result of a roundtable that offered a space for critical debate and reflection on the methodological and epistemological foundations that underpin existing feminist research on conflict, violence and peace. It took place at the International Studies Association Congress in Montreal in 2023. Taking stock of the variety of approaches (comparative, multimodal, theory-building) and theoretical standpoints of the roundtable speakers, we examined the (feminist) politics of knowledge production in academia. Participants discussed how ontological and epistemological assumptions shape what counts as (feminist) academic knowledge and what is considered to be possible in (policy) practice, and explored ways to enhance theory-building and comparative research, opportunities to create more equitable knowledge production practices and ideas on how to produce knowledge that can lead to policy change. We also reflected on positionality and reflexivity as well as the implications of engaging with sensitive stories and issues. We came together to dedicate space to voicing some of the personal, ethical and methodological challenges and dissonances of what it means to research violence in solidarity with our interlocutors.

We shaped the roundtable around the following questions, which we explore – in conversation – in the remainder of this article:

1. How do your ontological and epistemological assumptions shape what counts as (feminist) academic knowledge?
2. How can we shape our knowledge production practices for them to be more equitable? And for our knowledge to lead to change?
3. What have been your main personal, ethical and methodological challenges and dissonances in researching violence?

As scholars working in (post-)conflict settings – terms that we engage with and contest throughout our scholarship – we have been offered seminars, workshops and conferences on how to undertake our ‘fieldwork’, and ultimately how to produce and present knowledge. We read countless books that describe and prescribe what this work should look like, what form it should take and how we can measure whether it has been successful.^1^ Yet, as feminist scholars we are also aware of the complexities of what undertaking this research entails, both in terms of gathering, analysing and publishing what we are taught to refer to, and think of, as ‘data’.

Beyond data collection, we all come face to face with dissonance when it comes to researching violence. The challenges – personal, ethical and methodological – that we face often inspire uncertainty. Indeed, in Krystalli et al.’s recent chapter in the edited volume *Uncertainty in Global Politics*,^2^ they ask us to reflect on what it might mean to make friends with uncertainty. While this article was already nearing completion by the time we read their chapter, we have decided to take their question as guidance and inspiration as we collate and edit our reflections. Instead of seeking answers or truths that can be replicated according to the scientific method,^3^ we decided to tackle our uncertainties head-on, and in conversation. Thus, this article in itself is, for us, a way to embrace uncertainty, to make it our friend and to share these reflections with others in our discipline.

Indeed, the uncertainties and dissonances that we outline in this article reflect other discussions taking place *within* International Relations (IR). These critiques are sceptical towards Western, hegemonic ways of knowledge production itself.^4^ Authors engaging in this field draw on ‘various strains of post-structuralism, feminism, post-colonialism, and more recently, decolonial thought’ to create a room within the discipline for ‘distinct ways of knowing grounded in diverse lived experiences or ways of being in the world’.^5^ For Blaney and Tickner, the key lies in ‘[crafting] encounters across ontological difference and recognising the power at play in practices that convert Western realities into *the* reality’.^6^ Closer to our own research, Krystalli and Schulz shine a light on the epistemological hierarchies that exist in conflict research. These authors expose how conflict research rather myopically focuses on violence and suffering, and ask what we might see if we were to ask the question: ‘how does taking the practices of love and care seriously illuminate different pathways for understanding the remaking of worlds in the wake of violence?’.^7^ The intention of this article is not to summarise critiques of IR scholarship coming from within the discipline. With that said, we wish to situate it as a response to Blaney and Tickner’s prompt:

If worlds are made, the challenge that remains is to imagine creative and meaningful forms of reciprocity and collaborative practices that might be the basis for forging connection and mutually supportive relations across ontological difference.^8^

For us, then, making friends with uncertainty while doing research on conflict, violence and peace means taking seriously voices, spaces and temporalities that have often been dismissed because they do not fit the tidied-up narratives, rearranged in an academic format that requires offering causal pathways, clear results and filled ‘gaps’. It also means disengaging with the ‘singular world’ logics of IR introduced by colonial modernity and their disregard for alternative worlds^9^ and promoting a politics of ontology that privileges relationality, the pluriverse and active listening.^10^ Making friends with uncertainty enables us to amplify the significance of violent conflict to reveal how it manifests in bodies, emotions and sexualities and to inquire from spaces of intimacy and vulnerability often considered inconsequential because outside of negotiation tables, uniformed bodies and mediatised events. Spaces that blur the boundaries between the public and the private, spaces of the ‘tanteo en la oscuridad’^11^ in the search for meaning and ways to avoid the reproduction of the patriarchal grammar.^12^ In that search, we stumble upon advances and setbacks, shortcuts and detours that we consider important to document, to make visible. To do so, we take inspiration from Katherine McKittrick’s work on the Black method, as the practice of ‘bringing together multiple texts, stories, songs, and places’ that ‘involves the difficult work of thinking and learning across many sites, and thus coming to know, generously, varying and shifting worlds and ideas’.^13^

The uncertainties we speak about above also manifest clearly in our encounters with our interlocutors, the knowledge holders and the inevitable challenges of recognising the multidirectional power relations of our knowledge production practices. Against the required ‘objectivity’ of a distant and neutral scientific inquiry,^14^ feminist scholarship has advocated for the need to take responsibility for what we study and how we study it, to reveal our positionality and subjectivity and to centre doubts and contradictions.^15^ Why do we decide to select certain data and citations in our writing from all the material that we have collected? How do we ‘translate’ the experiences and visions of the world that have been confided to us? Are we misrepresenting those experiences and visions in our meaning-making processes?^16^ In what follows, we explore what it means to us as feminist researchers to centre uncertainty and to recognise the vulnerability and discipline that comes with it, but also the openings and possibilities offered by a research process that privileges the coexistence of multiple truths, of the ‘not only. . . but also’, and that is inevitably collective, relational and in constant transformation.

In what follows, we recreate the conversation we had in Montreal in 2023. We answer three questions that allow us to reflect on how we identify, navigate and even embrace uncertainty in our feminist research. In doing so, we pull from examples in our own lived experiences to assess how Western and hegemonic practices of knowledge production creep into our lives as feminist academics. Finally, we offer – by way of concrete examples – suggestions for how to think about, understand and disrupt a ‘singular world logic’ when it comes to producing knowledge with and about communities of which we are not part.^17^ Thus, in this article, we can expand beyond the original parameters of the roundtable to reflect on what the methodological dilemmas we identify may imply for the limitations of knowledge production more broadly.

1. How do your ontological and epistemological assumptions shape what counts as (feminist) academic knowledge?

Priscyll: Upon reflecting on this question, my first thought was that ‘landing’ into ‘feminist’ – and I would add, decolonial – knowledge ‘production’ meant trouble, and at times, pain. I have been troubled by how unaware I was about ‘how I came to know what I know’ and how I had (and still have) to ‘relearn’ and ‘undo’ so many of my assumptions. It has been painful because it has meant destroying my ontological and epistemological assumptions. It has meant not only trying to operate a change in how I conduct research but also in my affective relationships. And both, *feeling and knowing*, became intrinsically related.^18^

On the one hand, referring to oneself publicly as ‘feminist’ is sometimes impossible for the women with whom I conduct research. For some of them, this label means being tagged as a deviant, insurgent, revolutionary woman, implying that ‘producing’ knowledge from a feminist perspective, in practice, is highly risky.^19^ Therefore, I feel that I am very privileged, both to be able to name myself as a feminist in the public sphere (although not always) and also to have spaces of reflection with my interlocutors on *doing feminisms* in the everyday.

On the other hand, feminist knowledge per se is still not integrated into the mainstream discipline in which I work – mainly International Relations and War Studies. Accordingly, I feel that this way of thinking does not count as ‘knowledge’: on multiple occasions my research – even in environments where people should be interested in ‘gender’ – was recast as ‘militancy’ or ‘political activism’. Equally, working with women who are portrayed as ‘deviant’, ‘insurgent’ or ‘revolutionary’ means that even in feminist circles, the conversation sometimes became hard. For example, a lot of epistemological assumptions in Feminist Theories rely on antimilitarism as a core value and premise – almost as an ontological *certitude*^20^ – which can foreclose certain discussions.

Something that has been important in shaping my view of knowledge production is the belief that it needs to be *created in discomfort* with the current state of gender, class and race oppression, and to be collectively built. As Bilge^21^ reminds us about intersectionality, I believe that feminisms, too, are militant forms of knowledge. This position, assuming the destruction of the idea of the ‘expert’, has influenced and broadened what I believe counts as (feminist) academic knowledge. It allows for retaking the radicality of political knowledge production, and the refusal of the division between emotions, embodiment and research practices as important epistemological pillars about which to be curious. Ultimately, *feeling* our research, and integrating emotions into knowledge-building is also a refusal of the division between ontology and epistemology.

Nancy: My sociological research comes from my practice of international human rights law, and it is directly informed by it. With over a decade’s experience of working with and for human rights defenders (HRD) at risk, mainly in Latin America, I have been able to see a difference in the types of attacks, obstacles and challenges women HRD face. These challenges are even more pronounced when HRDs are also Indigenous, Afro-descendent or *Campesino* leaders. As a result, I wanted to explore their situation in more detail, equipping myself with more methodological and analytical tools than those provided by my legal training. Once I had decided to investigate the situation of Indigenous women leading the defence of human rights from abuses committed in connection to megaprojects in Latin America for my academic investigation – my DPhil in Sociology^22^ – my first instinct was to focus on the failures of the States in protecting them from the attacks targeted at them in reprisal for defending their communities, territories and rights. I came with two assumptions: (1) that an international legal human rights approach has mainly focused on the States’ responsibilities and on how to influence them as the main subjects of international law and (2) that a feminist posture believes in and works to advance all human rights for all women and girls. However, adopting a sociological research framework challenged my assumptions and helped me to move beyond a victimisation focus. Combining legal, sociological, feminist and intersectional studies, I have been able to acknowledge the victimisation of Indigenous women leaders in my research work but also to go beyond this approach to study their agency, leadership, mobilisation and impact.

Over the course of my research, Indigenous women leaders’ knowledge and experience became fundamental to my work. The learnings I gathered from their knowledge and experience became as important – or sometimes even more important – as those I gathered from academic literature. This is how the ‘braided action’ theoretical framework emerged from my investigation to explain my research question.^23^ It was only later that I realised that approaching my research from a non-hierarchical view that privileges the academic literature over the knowledge of Indigenous peoples (and Indigenous women leaders) was helping me to decolonise my knowledge and way of working. Indigenous women leaders were not only the protagonists of fundamental social mobilisations to protect their communities and rights but also became the protagonists of my research work and its policy impact, as I will explain in my response to the next question.

Maria: When I reflect on this question, I immediately think about how conflict and development scholarship distinguishes between theory and empirics, between expert and experiential knowledge, and how this is contrary to our ontological and epistemological assumptions as (decolonial, postcolonial, critical) feminist scholars. For the past 10 years, I have worked with and about Liberian and Burundian women’s organisations, documenting their activist discourses, practices and transnational alliances, and have written about those to challenge dominant theories on international norm production and diffusion.^24^ I have done so from my positionality as a privileged European academic researcher who can take part in panels, roundtables and academic publications that often have required me to make clear distinctions about what is theoretical knowledge, and what is empirical knowledge that illustrates my theoretical argument.

The presence of these discussions is per se an interesting indicator of the depth of the problem, but there is still a lot of work to be done to transform the pervasive hierarchies between what we consider as theory and what we consider as empirical material that illustrate our theorising. Nwando Achebe starts her book Female Monarchs and Merchant Queens in Africa with the words ‘*Whose histories, whose stories, whose archives?*’^25^. She does so to explain that researching and narrating African history and IR has historically and contemporarily been done through Western tools. That is, African peoples, and particularly women, are often seen as objects of knowledge production, and when they are considered producers of (experiential) knowledge, their knowledge is then ‘translated’ by the expert who will do the theorising. This is because experiential knowledge is understood as an expression of data,^26^ or as storytelling. But as Black feminists and decolonial feminists have explained, science and story are not discrete, rather, as feminists we know, read, create and feel science and story simultaneously.^27^ Recently, I listened to a conversation with professor Sabelo Ndlovu-Gatsheni who said that knowledge is made and emerges in struggle and that these types of knowledge cannot be as smooth and neat as if it was knowledge produced just for the sake of intellectual curiosity. In this way, Ndlovu-Gatsheni was also pointing out the epistemic violence of erasing different, yet legitimate, ways of understanding reality and ‘navigating’ it outside the intellectualised academic way privileged by Western thought.^28^

But Nwando Achebe’s question is not only a normative concern about who is a legitimate and authoritative subject of knowledge production but also an intellectual. This also highlights how the production of knowledge is intimately related to the (re)production of spatial hierarchies and it is deeply enmeshed with what happens in the world. There is an adoration of macro-theories and big ideas, but how we understand the world and think about the world is deeply embedded in the material realities in which we live. Indeed, the burden of demonstrating that you are bringing some theoretical findings to IR and conflict studies is bigger for those who want to theorise from data that does not come from the West. If IR does not build on our confusion when trying to understand events that are happening in a specific place from the bottom-up, and how these are linked to national, regional and transnational, multi-scalar processes, and doing so from the lived experiences of communities, then what’s the point?

Julia: I echo the above comments of my colleagues and add one more reflection. As I wrote my DPhil thesis, which then became my first book,^29^ I reflected on the research questions that I had asked, sitting in a library at the University of Oxford. In the final section of my book, I made a brief – but deeply important – acknowledgement: ‘My ability to research some of the country’s far-flung corners has only been made possible through the generosity of people like [my interlocutors]. On our many car, taxi, and bus rides, they have explained to me how Colombia works in practice. They have listened to my questions, and patiently helped me shape better ones’.^30^ I firmly believe in acknowledging that my feminist knowledge has been refined by the people I work with, those who embody feminist activism as a daily practice and way of shaping their lives.

Indeed, in my work about high-risk collective action in Colombia, El Salvador and Mexico over the past decade, one of my resounding experiences is that of being humbled. I am writing this section from the San Salvador airport, after leaving the 15th *Encuentro Feminista*, a regional meeting that brought together over 1300 feminists from all over Latin America and the Caribbean to meet, share and act in solidarity. As I leave the *Encuentro*, I am – now, unsurprisingly – once again reflecting on how I am going to reshape my research questions for my forthcoming book project so that they better reflect the ‘so what’ questions being asked not by other academics, but by the activists with whom I stand in solidarity.

Maria: Crucially, as feminists, we have refused to separate knowing and feeling, experiencing and knowing, and therefore, are more suited to engage not only with other forms of knowing but also with other *ways of being* in the world. However, while our feminist ontological and methodological positions have pushed us towards epistemic diversity and a relational understanding of knowledge production, we still grapple with the limitations of IR academic knowledge as inherently partial and limited because of its mind-world dualism.^31^ This unease comes from an acknowledgment that our very ontological commitments to intellectual knowledge generation and the ensuing knowledge production practices have their limits,^32^ as these contribute to the ‘othering processes’ of Indigenous communities, women organisations we work with, and local communities and their ways of life, which are incommensurable to Western forms of knowledge.

2. How can we shape our knowledge production practices for them to be more equitable? And for our knowledge to lead to change?

Julia: As an academic who has built a parallel career around advocacy and policy change, I often see myself as a translator. A translator between languages and worlds, between stakeholders and spaces. Much of my feminist policy engagement takes place through Ladysmith, a feminist research collective that supports international organisations to collect, analyse and take action on gender data. It was Ladysmith’s founders, Tara Cookson and Lorena Fuentes whose work about the unintended consequences of well-intentioned interventions inspired my approach to bridging academic and policy work.^33^

I bring their reflections to my academic work as well as my engagement with women participating in high-risk collective action around Colombia. One of the biggest obstacles to real change that I found was the difference between what a well-intentioned policy looks like in an international space, and how it plays out on the ground. For example, the Women, Peace and Security Agenda, which is so heavily featured in the design and implementation of the Colombian peace process, encouraged grassroots Colombian women to take the lead in peacebuilding initiatives in their communities. Yet, such activities can be incredibly dangerous work, particularly in areas where the dynamics of violence and armed conflict are in a process of reconfiguration.^34^ Putting the burden on women to engage in collective action can actually put them in grave danger and without the appropriate measures to guarantee their ongoing access to resources, let alone their physical security and that of their families.

The take-away message from much of my research is that the on-the-ground implementation of agendas (including Women, Peace and Security) needs to come from the bottom-up, as well as the top-down. As an academic, then, I take it as a responsibility to translate between these different spaces: making women’s issues make sense to the different actors involved who may not have a direct line of communication. By working with women at the grassroots level, I am afforded insights into how we can centre their needs into feasible and transformative policies that are context-appropriate. As I write in the prompt below, working to bring different voices and people with different levels of privilege and access into elite spaces is part of overcoming some of the ethical dissonances inherent to the work I do, as a Global North researcher working in the Global South. Ensuring that I can bring the knowledge I am generously gifted by my interlocutors to the spaces where decisions are made is a non-negotiable component of the responsibility that I have as a researcher.

Marie: Julia references something critical: that transformative change can only happen through simultaneous top-down and bottom-up processes. Feminists have long helped us notice those at ‘the bottom’ – the subaltern, marginalised and excluded – and asked us to work to shift power towards these communities to build more equitable and just worlds. My work has focused on those bottom-up processes, exploring how women impacted by violence have found innovative and creative ways to carve out different possibilities for themselves and their communities in the aftermath of war.

I think research can support this process of catalysing ‘bottom-up’ power *if* done with feminist, decolonial and non-hierarchical commitments. We are all aware of the critiques of the tremendously extractive nature of much social science research, especially research that focuses on war and its aftermath. But charting a new course for research within the constraints of the tenure, publication and grants ecosystem is hard, especially for junior or precarious scholars. In my own experience, what I’ve thought to be the most transformative parts of my research have rarely resulted in a publication or a measurable output. Thus, the incentives are stacked against these feminist innovations in the research process.

In my most recent work on the *Women’s Rights After War* project, which I run with Milli Lake and a collection of feminist researchers, we’ve strived to integrate collaborative, arts-based and participatory research into all phases of the research design, data-gathering and analytical processes. This has meant shifting how we spend money – such as, when we can, prioritising the financial security of all of the junior and Global South scholars on the project before we expect them to contribute to the research process. We make decisions about the project collaboratively amongst our organising team and have prioritised ensuring that all junior and Global South scholars on the project have ample publication opportunities as a result of their work. We’ve also integrated participatory workshops that are co-designed with artists and activist collectives in the contexts where we work. These workshops have been both data-generative (e.g. we’ve conducted short interviews with participants), but they’ve also been a holistic intervention in themselves. For instance, at an art workshop in Bogotá, Colombia last year, we collaborated with a photography collective (RADAR) and two Argentinian artists to bring women who had lost loved ones during Colombia’s war together to make art. The South-South connection between the Argentinian artists and the Colombian women established a powerful sense of solidarity that connected different experiences of grief and loss across contexts, and the art of making something beautiful out of their pain led one woman to exclaim, ‘this [workshop] was closer to justice than anything I’ve experienced in the courts’. We are proud of these elements of the project and feel strongly that they are part of feminist research praxis, and yet we also recognise that they haven’t been easy to implement, fund or execute.

Priscyll: I believe that the colonial, gendered and racist roots of academia are embedded in knowledge production – and the institutions that sustain it – and make it challenging to reach equity in practice. However, I also believe that it is possible to work towards a micropolitics of research that could, at the level of the bodies, the emotions and the ‘everyday politics’, work to bring changes. Like Julia, in researching violent contexts, I have tried to think about how I can contribute to making those changes. I would like to mention a few of them, which I think can also affect the way we, as researchers, influence policy change.

First, I think what could shape our practices differently is accepting that ‘fieldwork’ never really happens as planned: we should embrace failure^35^ and, maybe, become ‘bad researchers’ in the eyes of neoliberal academia.^36^ It is impossible, in war settings, to produce ‘neutral’ knowledge.^37^ Fieldwork in war and post-war also produces insecurities and risks, and to me, this has meant accepting that fieldwork is circular^38^ and that you never really come ‘out’ of it. Fieldwork is not a phase of research, but rather, it implies constant feedback and loops between methods and theory-building, between what is felt on the ground and what knowledge creation is. This constant connection and circular thinking – apart from helping us not to fall into the trap of progressive and cumulative knowledge production – also brings to the fore ideas on how to stay connected with what can actually be changed through policymaking.

Accepting the circularity of fieldwork also means that we can open the possibility of deeper conversations with the people we research with. As I have shared elsewhere, I think that adopting ‘friendship as methods’^39^ can change the ‘research temporalities’ by following the ‘pace of friendship building; slow, disruptive, and uncomfortable’.^40^ Engaging in friendship through research is not about ‘becoming friends’ with everyone we are researching with. It is about adopting an ethics of research that would take emotions as central tenets of knowledge production – I do not think we can change policies, or structural oppressive systems if we do not care, in the first place, about *why we do research* and *which emotions are circulating* in this action of researching. Friendship is political and affective; it can be a powerful vehicle of opposition to ‘scientific neutrality’, disembodied knowledge and pressure to ‘produce’. It allows for a space in researching war and peace, where we can feel that we take a stance for social justice and alter/transform power relations.

Then, to shape different knowledge production practices, we need to think about knowledge as collectively – and hopefully, creatively – *created*. Prioritising the ethics of friendship applied to research helped me to think about how I should engage in the long run and constant dialogue with the people participating in my research. This means thinking of research as a political conversation, a site for contentious and creative knowledge-building. This implies a constant fight against scientific rationality that assumes a hierarchical and clear distinction between ‘participants’ and ‘researchers’. Collective knowledge production invites us to think about ways of writing collectively and building long-term ‘transversal politics’ and coalition building.^41^ Researching with these views in mind means refusing to align with the *status quo* of knowledge production, which is not easy, as it asks us to constantly reflect on the projects we are proposing. This is especially true in violent settings.

I also want to add that when it comes to the policy implications of my research topic – related to the Disarmament, Demobilisation and Reintegration (DDR) processes, especially on women and feminised bodies in Colombia – it has been particularly challenging to make space for feminist knowledge as I outlined in the first question. The field of DDR is not keen on integrating radical politics and, most of the time, it perpetuates the *status quo*, which sustains the patriarchal, classist and racist social order. ‘Gender’ has been highly ‘mainstreamed’ by the international peacebuilding apparatus,^42^ but in reality, the lives of ex-combatants I work with have hardly been improved. This is especially true for women, girls and LGBTIQ+ people. Emotions, affects and embodiment are not framed as political subjects in DDR programs, which mostly follow a public-private divide.^43^ Given their overall focus on the technical and economic dimensions of DDR, those programmes fail to acknowledge the political transformations that occur between an armed struggle and the normalisation of combatants into ‘civilian’ – a status equally bound by norms and values attached to normative views of citizenship^44^.

Nancy: As I mentioned above, in my academic research, I have explicitly embraced the fact that my work comes from the practice of human rights and continues to be connected to it. For this, I have built on the sociological praxis method as one of the main pillars of my methodological orientation.^45^ This research-action approach looks at the problem of researching reality to transform it. This method is concerned with scholars’ responsibility to interpret realities’ transformation to derive adequate data that helps build the future. Thus, ‘. . .we cannot ignore the social, political and economic impact of our work’.^46^

In this vein, I have always communicated the notion of ‘sociological praxis’ from the outset of preliminary contacts with Indigenous women leaders across the Americas. From the moment I introduce myself and the study (its aim, nature and methodology), I am explicit about my research-action orientation. Some of the Indigenous women leaders who shared with me their knowledge and experience expressed their interest in participating in my study only after they learned about the ‘sociological praxis’ approach I use. They understood this methodological approach as a way to eliminate the possibility of repeating previous experiences where they felt that academic projects had ‘a colonialist approach’ oriented towards ‘intellectual extractivism’.

In the field, the application of the sociological praxis approach was reflected in a constant and mutually beneficial knowledge exchange process. I was not only focused on the future of this work but also on the present moment and on how my legal and international background could be useful for their most immediate concerns. For example, before one of the research interviews, an Indigenous leader had been detained and was incommunicado; instead of interviewing the Indigenous women leader, we wrote an ‘urgent action’ together and sent it to all our contacts, raising the concerns and seeking support. I had drafted many ‘urgent actions’ in my previous practice, and this was a skill I used, applying my methodological orientation. This is a tangible way to dismantle perceptions of asymmetric power relationships between the researcher/interviewer and the interviewee/s. This method has been key to building trust with people who have learned to distrust outsiders. It has also been a way to express empathy to women, men and communities that have endured decades of human rights violations and victimisation, whose accounts have often been met with disbelief or have been ignored, discredited or misused.

This way of working, however, may raise concerns about my critical distance or my dispassionate analysis. There is indeed a risk that the critical distance needed for sociological research and analysis is compromised with the idea of making visible these agents of change who have faced wrongdoing. There is the risk of ‘ethnographic seduction’.^47^ Yet, believing my interlocutors’ accounts and generating an ethical dynamic of knowledge exchange is not the same as analysing data uncritically. The combination of several qualitative methods and approaches in my academic work prevents me from accepting accounts uncritically for the analysis. In this regard, for example, I also use public sociology, life-history sociology and process-tracing methods, among others.

In this vein, another key pillar of my methodological orientation is public sociology. A study using this approach means that it is relevant in ‘. . .both the academia and the public sphere. . .’.^48^ This approach builds on the ‘Webster windmill of public sociology’, which responds to ‘the winds’ of social, political, legal and economic change and not only to a theoretical interest.^49^ Applying this methodological orientation also means that my findings should help to influence policy and institutions. As Julia discussed above, in this way, my research-action approach extends from the most immediate context of the Indigenous women leaders to other spheres of influence that can impact their situation. In this regard, for example, I have presented aspects of my findings in meetings and conferences, and I have also made submissions to domestic and international institutions, both the United Nations and the Inter-American system of human rights. My research and Indigenous women’s concerns and experiences are reflected, for example, in the 2022 report of the United Nations Special Rapporteur on the Situation of HRD, who issued a report on the occasion of the 25th anniversary of the UN Declaration on this matter: ‘Success through perseverance and solidarity: 25 years of achievements by human rights defenders’. I recognise that for knowledge to produce policy change, we should recognise the different levels of influence, the collective nature of the effort and the importance of the concrete experience of (Indigenous) women, as agents of change.

Maria: Like Nancy, I have worked following research-action and participatory-action methods that privilege the active involvement of people with lived experiences as co-researchers to generate new knowledge and act on findings to improve their lives. This, of course, contrasts with the analytical distance required by the discipline, that risks adopting a position of superiority over those people whose lives, experiences and feelings we write about. The analytical process might consist of seeking (or even creating) coherence in the narratives of those lives to provide better academic arguments. But lives are never coherent, they are contradictory and fluid. Facing the uncertainties and vulnerabilities, we discussed in the Introduction during and after fieldwork is a *strength* and a necessity when we want to develop reflexivity, criticism and responsibility in the construction of knowledge.

For example, Priscyll and I have conducted and co-produced research with a group of Colombian feminist musicians through participatory-action methods to understand how the arts provide bottom-up opportunities and spaces for healing after violent conflict for marginalised communities.^50^ One key aim of the project was to forge research that broke with the binaries between ‘the researcher and the researched’ and produced co-created knowledge. Instead of being extractive, we wanted to support the group to reflect on their common past and their strategy for their future. For us, both the ethos and the methods of the project sought to defy the research conventions of the scientific world, whereby an individual researcher can explain and trace causal mechanisms of a complex socio-political phenomenon. This is a fallacy, and thinking and feeling from the corporeal – from the body – is necessary if we are to produce knowledge outside of the confines of the logic of oppression and imposition of Western categories of analysis.^51^ We privileged data collection methods such as body mapping and long-lasting, multi-sited WhatsApp conversations and ‘friendships’^52^ that recognise research as a collective, long-term process of community and encounter, of approximations and fluidity, of the impossibility of certainty. As such, we recognise that the field cannot be a place ‘out there’ where you go and from where you come back, an ‘other’ space and time that can be ‘instrumentally penetrated and evacuated’.^53^ Centring the body and emotions – and their potential for opening (difficult) conversations – is how I believe feminist research that produces transformative knowledge and practices should be done.

In another project that seeks to understand collective resilience after episodes of extreme violence in the Democratic Republic of Congo, I work together with a local organisation specialised in place-based Indigenous cosmologies to organise Word Trees. The Word Tree is a traditional Central Africa community discussion tool in which people gather under the shadow of a tree to discuss community issues through storytelling. The aim is to open spaces to recover memories and experiences, and to build a common narrative and community solidarity. There is no map or pre-existing route^54^ to follow when doing feminist and decolonial research in conflict settings, but giving a central space to vulnerability, storytelling, collaboration and uncertainty has been important to me. Finding openings with others, rather than fixed conclusions as an ‘expert’ researcher, is how I believe research can bring about change.

In sum, the five of us, in our different feminist approaches and methodologies, are all committed to critical thinking and emancipation, to ‘stand apart from the prevailing order of the world and ask how that order came about’ rather than to ‘problem-solve’.^55^ The use of feminist methodologies and methods enables research subjects to take part in knowledge production processes as experts, but at the same time, I, Maria, am left wondering whether this research can integrate the knowledge produced in an Indigenous knowledge system into a Western academic one, or whether we are complicit in the reproduction of our own and limiting knowledge frameworks.^56^ How does one obtain informed consent to transpose and translate knowledge to another knowledge framework? What is there to lose? The challenges and dissonances in feminist research on conflict, violence and peace are many.

3. What have been your main personal, ethical and methodological challenges and dissonances in researching violence?

Julia: Maria finished the last section with questions, and began to reflect on some of the dissonance she feels in her feminist academic practice. I too have often found a certain level of dissonance in my research. One concrete example that comes to mind is the dissonance I experience between planning for research and actually undertaking it in violent contexts. While on paper – for example, in ethics protocols – it can sometimes become formulaic to write about how I will design my research to avoid retraumatising participants who have experienced violence, in practice this is not as clear-cut. For example, while I usually design interview guides to be semi-structured in a way that creates room for interlocutors to modify our conversations according to what they want to share with me,^57^ I find that often they *want* to talk about grief, loss, anger and pain. When working with *madres buscadoras* in Mexico, for example, I was careful to ask about their participation in collectives, rather than about their experiences of losing a loved one. Indeed, my ethics protocol required this approach. Yet without fail, each woman wanted to talk to me about her missing son or daughter or husband, to show me photos of them, to talk about what their favourite food was or what sports they liked to play.^58^ The blurred lines between not wanting to make a spectacle out of violence yet also facing situations where interviewees cope by sharing their intimate and painful moments, can be uncomfortable and present ethical challenges.

I find that in practice, some interviewees in vulnerable situations are looking for an opportunity to create an intimate tie. This is particularly the case when they have no access to psychological support, yet carry heavy grief loads or feelings of burnout. For example, in a digital data collection project I recently finished with 100 high-risk women leaders across Latin America, I was surprised that despite creating an online questionnaire to facilitate privacy when sharing information, many of the women wanted to arrange phone calls with my research assistants and me. This was after they had finished the interview; they wanted to talk – sometimes for extended periods of time – about the information they had already shared. My research assistants and I discussed the concept of *desahogo* and what it means when interviewees simply want someone safe to talk to about the experiences that they cannot share with others in their personal lives.^59^ For this reason, I truly enjoyed Priscyll’s piece on friendship in research, as it resonated with many of the interactions I have with women in Mexico, Colombia and El Salvador.

Relatedly, I often find myself reflecting on what it means to be a story sharer, one who tells stories that are not my own. Indeed, in our ethical protocols and within certain journals (feminist journals, in particular), we are encouraged to reflect on power differentials with our interlocutors, and how we will aim to overcome or mitigate these. Although I too write these reflections, I am not always convinced that these dynamics are ‘overcomeable’, at least not simply because we state that we have considered them. Instead, I find that balancing these often-inevitable dynamics is a much more active practice; sitting and having tea with someone, taking the time to chat with them beyond the parameters of the interview, modulating language and accent so that we can understand one another. I loved reading about the Word Trees in Maria’s reflection and wonder if this is a method that I have – completely unintentionally – used myself, for example, when I conducted group interviews under mango trees in La Guajira. The idea of needing to move beyond unbalanced power dynamics is not necessarily the end goal for me. Rather, a more ethical practice relates to bringing empathy and humanity to the way that we interact with people: not as research subjects, but as interpreters between worlds that are made different by a whole host of power dynamics. Marie Berry’s words on the humanity of being a researcher resonate with me. She writes about ‘[embracing] the persistent discomfort’ of relating stories that do not belong to her while also respecting and honouring the trust that is placed upon her. For her, the trust that her interlocutors give her ‘affirms my trust in the power and importance of sharing [their stories]’.^60^ Particularly in violent contexts, where there are indeed risks of sensationalising stories or experiences – becoming a voyeur into a scary or dangerous world – encountering the balance of what it means to engage in ethical research involves not only what we say we’re going to do, but also how we do it on a consistent, daily basis.

Maria: Julia, I completely feel that dissonance between planning for research, filling out all these ethic protocols designed to protect our employers against liability, and then actually doing the research from a serious feminist ethics of care towards our interviewees and colleagues in every research encounter. In my research diary, I always make notes about how I felt during an interview, a Word Tree event or a focus group. Sometimes I feel too much empathy, other times I wonder if I feel too little, which destabilises me. Sometimes I feel at ease, and other times I am left depleted, or angry, or disappointed. For me, this is an ethical feminist practice insofar as it breaks with the binaries of reason/emotion or body/mind and recognise the potential of the body and emotions as a site of fieldwork and data collection and analysis. The (dis)comfort I feel during the research process and its exploration gives us the opportunity to remind ourselves that the knowledge we seek to produce with our research is inevitably partial, and inevitably subjective. In a discipline in which the hegemonic value is associated with categorical and clear-cut statements and conclusions, being open to hosting uncertainty and vulnerability is scary and exhausting. Being genuinely reflexive and conscious of the conflictual dynamics in which we can exert epistemic violence is, as Enloe pointed out, not easy, nor comfortable.^61^

I still think about the dissonance that is born from the disconnect between what our interlocutors give to us, confide in us and what we as academics can give back. This concern has preoccupied me since my first days as a PhD student. One of my first encounters with a group of women activists in Burundi ended with an elderly woman holding both of my hands and telling me: ‘Now that you know our story, we are friends’. Those words continue to resonate in my head, as I understood that these women had certain expectations as to how (me) telling their story to an international audience could help in mobilising activists and funds. This dissonance continues during the writing process. The feminist ethnographer Judith Stacey pointed out that inequality, exploitation and even treason are endemic in ethnographic work.^62^ On the one hand, there is a fear of writing in a way that respects and cares for our interlocutors. On the other, there is a recognition that a certain level of exploitation is somewhat inescapable. While my academic career has clearly benefited from writing – with care and in solidarity – with women activists in Burundi, I cannot see how my ‘friendship’ has benefited them.

Acknowledging the constant presence of these dissonances and permitting ourselves to embrace them is essential to take seriously marginalised voices and to displace if at least for a moment, Western hegemonic ways of producing and disseminating knowledge in which scientific objectivity requires foregoing embodied and sensory experiences, emotions and feelings as important sources of data.

Nancy: Like Julia and Maria, I find engaging in research in contexts of extreme violence very challenging for so many reasons. In the specific cases of Indigenous women-led mobilisations defending rights and territories from abuses linked to megaprojects, extreme violence is exacerbated by historical patterns of oppression, such as racism, structural obstacles, including impunity and inequality, and violence specifically targeted at these women in reprisal for their legitimate actions in defence of human rights.^63^ The duty of care plays a central role in addressing such contexts. It needs to be very well-thought-out from the outset, towards the participants of my work and towards me as the person researching in such contexts. Although we often see these leaders as activists or HRD, their leadership is much more complex, as it involves their lives, their existence and their subsistence as individuals and as people.^64^ In this vein, such a duty of care should be reflected in the comprehensive ethical protocol that includes detailed security plans, preventive actions and solidarity support. The right and principle of free, prior and informed consultation and consent (FPIC) developed to protect and guarantee Indigenous rights and territories should be applied as a core ongoing principle to any research activity involving Indigenous peoples. Some Indigenous women leaders, their organisations and communities have developed their FPIC protocols, building on their ancestral ways and laws; others specifically refer to the United Nations Declaration on the Rights of Indigenous Peoples^65^; and, in some countries, such as Canada, specific ethical guidelines must be followed in this regard.^66^ Following ethics protocols like these is an additional important element to addressing challenging research contexts where Indigenous women leaders and their communities are the experts.

Priscyll: In my research in war or post-war settings, I have felt several methodological challenges, as my colleagues discussed above. In this context, dissonance is omnipresent. In fact, I believe that it is important to remain in this discomfort, as it pushes us to constantly question, ethically, what kind of research we aim to do and how we aim to transform violent realities.

What I feel feminist perspectives on methods *do to research* is that they create a continuous reflective path to be more creative methodologically, constantly contesting the terms imposed by an androcentric and colonial institution such as the university. For example, the *time* and *space* of research in (post)war settings never take place at the same pace as in academia. The neoliberal academia pushes for results, emotionless efficiency and disembodied productivity. Building trust and constructing long-term relationships with participants in violent settings take time and emotional space. If we aim to destructure the extractivist research apparatus of academia, time becomes central to open long-standing conversations and possible projects with the participants of research.

I feel that once I accepted failure and discomfort within my research practices – failure to adhere to the scientific and strict methods I was asked to perform – I actually started to create and write differently. I have also begun to think about forms of knowledge production with the people I have worked with.^67^ However, one of my main challenges, methodologically, has been that pushing alternative, art-based and creative methods always comes with a struggle against the dryness of academia and the emotional labour that it entails – which again, does not go with the time frames of our institutions and funders.

When reflecting on the word ‘dissonance’ for this piece, I looked in the dictionary and found that it was defined as a ‘lack of agreement’, an ‘inconsistency between the beliefs one holds or between one’s actions and one’s beliefs’. This definition puzzled me as I began to respond to this question, for two reasons. First, I realised that I am constantly in a state of dissonance – with myself, my beliefs and with the situations – while working and researching in/through violent contexts. And second, because I have trouble considering that the inconsistency is necessarily something ‘bad’. I wonder if dissonance actually holds an interstice where power, violence and other systematic oppression can be challenged and tackled. If this is the case, embracing constant dissonance, and working through and against it, might be a more fruitful way of conducting critical research.

One of the major dissonances I have experienced was during my work with perpetrators of violence, especially with people with whom I do not align ideologically, and who have committed crimes against humanity. In my experience researching paramilitary violence, I was troubled by several mechanisms that I have adopted to process the narratives of violence, to make sense of the lines between research and criminality testimonies, and to ‘protect’ myself from emotional breakdown. For example, with several practitioner colleagues, we have discussed the use of *humour* to process the narratives of violence and torture we were exposed to. But, isn’t it a dissonance to use humour in that context? While working with male paramilitaries in a detention centre in Colombia, I felt at times that I did not know who was interviewing whom – after all, they had been trained in war and torture techniques. I felt anger and fear – especially as one of the few women there. But I also felt puzzled because, sometimes I would realise who I was talking to only after I came out of the centre, and learned about the war crimes they committed. And the dissonance arose when I realised that, before knowing those facts, I had not felt any discomfort during the conversation. While these experiences humanise the perpetrators of violence, they also pose several questions about the ethics and emotionality of conducting research with them and about their violence. I have not resolved these contradictions.

Marie: When I read the words of my fellow panellists, I find myself nodding, repeatedly. What a joy it is to share intellectual space with each of them. Julia’s recognition of the dilemma that emerges when our research interlocutors share the stories *they want* to share – not the ones *we want* them to – resonates powerfully. I remember while conducting research in Bosnia for my first book,^68^ I met dozens of women who told me they would answer whatever questions I had prepared ‘*after I tell you about how I was raped*’.” The first few times I heard these words I was stunned, having carefully generated an interview protocol that focused on their experiences of power and civic engagement during and after the war. But before long, I came to the uneasy understanding that my presence as an active, empathetic listener allowed some women to feel that their horrific experiences were not being ignored or forgotten – that my role was less as an interviewer and more as a witness. This early experience transformed how I think about myself as a researcher and has motivated me to learn as much as I can about trauma-informed practices, embodiment and care so that I have a broader – yet still incomplete and imperfect – set of tools with which to respond in similar contexts.

Maria also articulates something that haunts me often: the powerful tension between what researchers take versus what we actually give back. All of us have experienced this feeling of impotence, that we can’t actually do much to change the lived conditions of people’s lives through our research. These days, I find myself trying to find small, imperfect ways of doing just that – shifting the material conditions of peoples’ lives, or amplifying their boldest desires through whatever resources I may have. I have tried to build programming alongside my research projects, convening initiatives that support women-identified activists or that can provide direct support in urgent situations to activists. All of these initiatives are insufficient and imperfect, but they reflect an attempt to hack the university and research systems in ways that can begin to whittle down the extractive imbalance of research processes.

For me personally, my biggest challenge in researching violence has been the way that my professional work has collided with my personal life. My life has been profoundly shaped by violence – some of my closest loved ones have had lives altered and ruined by the legacies of war and trauma. What initially seemed (even to me) like a professional interest in understanding how women survive after the war actually revealed itself to be a deeply personal one. My challenge since this realisation has been how to hold both stories at the same time – the analytical ones that comprise my professional research agenda and the personal ones that shape my entire world. What I know by listening to both stories is that trauma is a tentacular beast with many limbs and appendages that morphs and shapeshifts until it is transformed.

## Conclusion

As Priscyll concluded above, we have not resolved the contradictions and dissonances discussed in this article. Indeed, as she and Maria compellingly suggest, perhaps it should not be our goal to resolve them, but rather to openly acknowledge and critically reflect upon them – to permit ourselves to embrace them. In doing so, we can open ourselves up to making friends with uncertainty, and use it as a way to motivate the deepening of what we consider our feminist academic knowledge and praxis. This ‘friendship’ is necessarily a research practice, rather than a problem-solving or box-ticking exercise. Indeed, while we reflect on ontological, epistemological and methodological questions throughout, we also hold space for the very real and pressing material contexts of precarity and neoliberalisation of academic institutions as shapers of knowledge production processes and output.^69^

In this article, through feminist discussion, we have made three main contributions. First, the production of knowledge within disciplinary boundaries, and in particular, IR, is closely related to the discipline’s history of positivism and exclusion. Only through interdisciplinary work can we continually entangle and disentangle varying narratives and tempos that together, invent and reinvent knowledge, privilege collaboration and discredit ethnic absolutism.^70^ As McKittrick quoting Sylvia Winter indicates, discipline is an empire^71^ and critical knowledge must be produced through interdisciplinarity and a broad conception of science that cherishes uncertainty, collaboration and storytelling, as well as a refusal to engage in a division between ontology and epistemology.

Second, Black feminist studies and anticolonial thought offer methodological practices that are well suited to sit with uncertainty. They give us permission to read, live, hear, groove, create and write across a range of temporalities, places, texts and ideas that build on existing liberatory practices, and to undo hierarchies of knowledge that determine which data is considered good scientific data.

Third, building bridges between feminist scholars and feminist activists requires alternative ontologies, epistemologies and communities based on attentive and active listening, questioning and unlearning, and leaving behind certainty to find the common threads that link our theory and praxis.^72^ In this article, then, we highlight that embracing uncertainty means accepting incommensurability and heterogeneity, as well as a shift away from the urge to accumulate knowledge and towards sitting with ‘multiple-word realities’.^73^ Indeed, we must do so, because any serious attempt to decolonise knowledge production will necessarily involve paying close attention to the nuanced and complex oppressive systems based on gender, sexualities, migration, poverty, and religion and how these impact ‘how we know’.^74^ That is, we argue that there can be more value in paying attention to, focusing and reflecting on encounters in the field (the process), rather than simply following the scientific method and/or myopically accumulating data for data’s sake (the result). As Julia wrote above, it was through spending time with her interlocutors during research – and listening to them – that she was able to shape better, more appropriate, more important research questions.

Before concluding, we want to acknowledge that as we finalise this article (and perhaps even more disturbingly, as we revisit this article 6, and then 10 months after submission), we are witnessing genocide unfold in Gaza. While none of us directly study this particular conflict, as feminist researchers writing about dissonance, uncertainty and dilemmas, it would be remiss not to mention the complicated emotions that emerge as we observe from afar some of the worst iterations of what conflict and violence can bring to communities. As we outline above, the intention of this article is not to conclude, nor make prescriptive recommendations about what ‘is to be done’. Yet, as Priscyll wrote, the visceral closeness of writing about ethical dilemmas as feminist conflict researchers and the *realities* of watching them indeed presents uncertainty, and perhaps a type of uncertainty with which we would rather not become friends.

We certainly do not want to equate witnessing genocide with experiencing it. Nor do we want to obfuscate the (gendered) horrors that wage on in other parts of the world: in Ukraine, Sudan, Iran, Myanmar, Latin America and so many other places. However, we acknowledge that there is a level of dissociation that necessarily comes with reading the articles, letters and posts on social media, and then continuing with business as normal in our university environments, particularly when ‘business’ involves writing and teaching on feminist approaches to conflict and violence. We hope that conversations like these can be spaces to engage with how to navigate profound and painful dilemmas, and how to voice our feminist knowledge particularly when the need for peace is immediate and urgent. We know that they are not solutions, nor do we see them as ivory-tower spaces for self-congratulation. They are, however, an earnest and heartfelt attempt at knowledge-sharing in the spirit of pursuing, supporting and indeed urging feminist peace.

## Notes

1. Patrick T. Jackson’s work discusses in detail the problems that arise when we ascribe to a singular or ‘scientific’ method within international relations and global studies. Patrick T. Jackson, *The Conduct of Inquiry in International Relations*, 2nd ed. (London: Routledge, 2016).

2. Roxani Krystalli, Shambhawi Tripathi and Katharina Hunfeld, ‘Making Friends with Uncertainty: Hopeful Futurities in Telling Stories About Global Politics’, in *Uncertainty in Global Politics*, ed. Anastasia Shesterinina and Miriam Matejova (London: Routledge, 2023).

3. Jackson, *The Conduct of Inquiry in International Relations*.

4. David Blaney and Arlene Tickner, ‘Worlding, Ontolgical Politics, and the Possibility of a Decolonial IR’, *Millennium* 45, no. 3 (2017): 293–311.

5. Ibid., 294

6. Ibid.

7. Roxani Krystalli and Philipp Schulz, ‘Taking Love and Care Seriously: An Emergent Research Agenda for Remaking Worlds in the Wake of Violence’, *International Studies Review* 24, no. 1 (2022): viac003.

8. Blaney and Tickner, ‘Worlding, Ontolgical Politics, and the Possibility of a Decolonial IR’.

9. Ibid.

10. Rama Sala Dieng, ‘Speaking Out, Talking Back? African Feminist Politics and Decolonial Poetics of Knowing, Organising and Loving’, *Review of African Political Economy* 51 (2024): 1–13.

11. María Lugones, *Pilgrimages/Peregrinajes: Theorizing Coalition against Multiple Oppressions* (Lanham, MD: Rowman & Littlefield Publishers, 2003).

12. Sarah Ahmed, *Living a Feminist Lift* (Durham, NC: Duke University Press, 2017).

13. Katherine McKittrick, *Dear Science and Other Stories* (Durham, NC: Duke University Press, 2021), 5.

14. Sandra Harding, ‘Introduction: Is There a Feminist Methodology’, in *Feminism and Methodology*, ed. Sandra Harding (Bloomington, IN: Indiana Universtiy Press, 1987), 1–14.

15. Donna Haraway, ‘Situated Knowledges: The Science Question in Feminism and the Privilege of Partial Perspective’, *Feminist Studies* 14, no. 3 (1988): 575–99.

16. Scholars like Linda Tuhiwai Smith, *Decolonizing Methodologies: Research and Indigenous Peoples* (Dunedin, New Zealand: University of Otago Press and Zed Books, 1999) offer valuable insights with which to shape reflections about extractive research and Indigenous and decolonial methods.

17. Here, discussions of the ‘insider/outsider conundrum’ are generative in terms of understanding researchers’ positionalities vis-à-vis the communities in which they work. See Nicola Savvides et al., ‘Journeys into Inner/Outer Space: Reflections on the Methodological Challenges of Negotiating Insider/Outsider Status in International Education Research’, *Research in Comparative and International Education* 9, no. 4 (2014): 412–25; Swati Parashar, ‘Research Brokers, Researcher Identities and Affective Performances: The Insider/Outside Conundrum’, *Civil Wars* 21, no. 2 (2019): 249–70.

18. Priscyll Anctil Avoine, ‘Insurgent Peace Research: Affects, Friendship and Feminism as Methods’, *Conflict, Security & Development* 2, no. 5 (2022): 435–55.

19. Julia Zulver, *High-Risk Feminism in Colombia: Women’s Mobilization in Violent Contexts* (New Brunswick, NJ: Rutgers University Press, 2022).

20. Priscyll Anctil Avoine and Bénédicte Santoire, ‘WPS Agenda and Women’s Armed Resistance (Im)Possible Agency?’ (paper presented at the International Studies Association, Montreal, QC, Canada, 2023).

21. Sirma Bilge, ‘Le blanchissement de l’intersectionnalité’, *Recherches Féministes* 28, no. 2 (2015): 9–32.

22. Nancy Tapias Torrado, ‘Indigenous Women Leading the Defence of Human Rights from the Abuses by Mega-Projects in Latin America, in the Face of Extreme Violence’ (DPhil Thesis, University of Oxford, 2020).

23. Ibid.

24. Maria Martin de Almagro, ‘Lost Boomerangs, the Rebound Effect and Transnational Advocacy Networks: A Discursive Approach to Norm Diffusion’, *Review of International Studies* 44, no. 4 (2018): 672–93; Maria Martin de Almagro, ‘Producing Participants: Gender, Race, Class, and Women, Peace and Security’, *Global Society* 32, no. 4 (2018): 396–414.

25. Nwando Achebe, *Female Monarchs and Merchant Queens in Africa* (Columbus, OH: Ohio University Press, 2020).

26. McKittrick, *Dear Science and Other Stories*, 36.

27. Silvia Tamale, *Decolonization and Afro-Feminism* (Montreal, QC: Daraja Press, 2020); Dieng, ‘Speaking Out, Talking Back? African Feminist Politics and Decolonial Poetics of Knowing, Organising and Loving’.

28. Jarrad Reddekop and Tamara Trownsell, ‘Disrupting Anthropocentrism Through Relationality’, in *International Relations in the Anthropocene: New Agendas, New Agencies and New Approaches*, eds. David Chandler, Delf Rother and Franzisca Müller (Springer International Publishing, 2021), 441–58, specifically 445.

29. Zulver, *High-Risk Feminism in Colombia: Women’s Mobilization in Violent Contexts*.

30. Ibid.

31. Jackson, *The Conduct of Inquiry in International Relations*. 35-6.

32. Tamara Trownsell, ‘Fostering Ontological Agility: A Pedagogical Imperative’, in *Signature Pedagogies in International Relations*, ed. Jan Lüdert (Bristol: E-International Relations Publishing, 2021).

33. Lorena Fuentes and Tara Cookson, ‘Counting Gender (in)Equality? A Feminist Geographical Critique of the “Gender Data Revolution”’, *Gender, Place & Culture* 27 (2019): 881–902.

34. Julia Zulver, ‘The Endurance of Women’s Mobilization during Patriarchal Backlash: A Case from Colombia’s Reconfiguring Armed Conflict’, *International Feminist Journal of Politics* 23 (2021): 440–62.

35. Katarina Kušić and Jakub Záhora, ‘Introduction: Fieldwork, Failure, IR’, in *Fieldwork as Failure: Living and Knowing in the Field of International Relations*, eds. Katarina Kušić and Jakub Záhora (Bristol: E-International Relations Publishing, 2020), 1–17.

36. Priscyll Anctil Avoine and Camille Boutron, *‘L’épistémologie féministe à l’épreuve d’un objet surinvesti. Enquêter auprès des femmes ex-combattantes dans le “post-accord de paix” colombien’*, *Annales de Géographie* 6, no. 742 (2021): 20–46.

37. Melanie Richter-Montpetit, ‘Militarized Masculinities, Women Torturers, and the Limits of Gender Analysis at Abu Ghraib’, in *Researching War: Feminist Methods, Ethics and Politics*, ed. Annick Wibben (London: Routledge, 2016).

38. Anctil Avoine, ‘Insurgent Peace Research: Affects, Friendship and Feminism as Methods’.

39. Lisa M. Tillmann-Healy, ‘Friendship as Method’, *Qualitative Inquiry* 9, no. 5 (2003): 729–49.

40. Anctil Avoine, ‘Insurgent Peace Research: Affects, Friendship and Feminism as Methods’, 443.

41. Annick Wibben and Amanda E. Donahoe, ‘Feminist Peace Research’, in *The Palgrave Encyclopedia of Peace and Conflict Studies*, eds. Oliver Richmond and Gëzim Visoka. (London: Springer International Publishing 2020), 397–405.

42. Camille Boutron, ‘Engendering Peacebuilding: The International Gender Nomenclature of Peace Politics and Women’s Participation in the Colombian Peace Process’, *Journal of Peacebuilding and Development* 13, no. 2 (2018): 116–21.

43. Sanne Weber, ‘From Gender-Blind to Gender-Transformative Reintegration: Women’s Experiences with Social Reintegration in Guatemala’, *International Feminist Journal of Politics* 23, no. 3 (2021): 396–417.

44. Luisa María Dietrich, *La Compañera Guerrillera as Construction of Politicised Femininity: A Comparative Study of Gender Arrangements in Latin American Insurgencies and New Paths for Gender Responsive Demobilisation of Combatants* (Universität Wien, 2017); María Estrada-Fuentes, ‘Affective Labors: Love, Care, Solidarity in the Social Reintegration of Female Ex-Combatants in Colombia’, *Lateral*, 25 October 2016. Available at: https://csalateral.org/issue/5-2/affective-labors-love-care-solidarity-colombia-estrada-fuentes/.

45. Orlando Fals Borda, ‘Por La Praxis: El Problema de Cómo Investigar la Realidad para Transformarla - 1978’, *Espacio Abierto* 31, no. 1 (2022): 193–221.

46. Ibid., 193.

47. Antonius Robben, ‘Ethnographic Seduction, Transference, and Resistance in Dialogues About Terror and Violence in Argentina’, *Ethos* 24, no. 1 (2004): 71–106.

48. César Rodríguez-Garavito, ‘Investigación Anfibia’, 2013. Available at: http://www.dejusticia.org/files/r2_actividades_recursos/fi_name_recurso.304.pdf.

49. M. Burawoy, ‘Southern Windmill: The Life and Work of Edward Webster’, *Transformation: Critical Perspectives* 72/73 (2010): 1–25.

50. Maria Martin de Almagro, Priscyll Anctil Avoine and Yira Miranda Montero, ‘Singing Truth to Power: Transformative (Gender) Justice, Musical Spatialities and Creative Performance in Periods of Transition from Violence’, *Security Dialogue* (2024). https://doi.org/10.1177/09670106241232265.

51. Elizabeth L. Sweet and Sara Ortiz Escalante, ‘Bringing Bodies into Planning: Visceral Methods, Fear and Gender Violence’, *Urban Studies* 52, no. 10 (2015): 1826–45.

52. Anctil Avoine, ‘Insurgent Peace Research: Affects, Friendship and Feminism as Methods’.

53. Maya J. Berry et al., ‘Toward a Fugitive Anthropology: Gender, Race, and Violence in the Field’, *Cultural Anthropology* 32, no. 4 (2017): 540.

54. Ruth Behar, *The Vulnerable Observer: Anthropology That Breaks Your Heart* (Boston, MA: Beacon Press, 1996); Yasmin Gunaratam and Carrie Hamilton, ‘Introduction: The Wherewithal of Feminist Methods’, *Feminist Review* 115, no. 1 (2016): 1–12.

55. Robert W. Cox, ‘Social Forces, States and World Orders: Beyond International Relations Theory’, *Millennium* 102, no. 126–155 (1981): 129.

56. Maria Eriksson Baaz and Maria Stern, ‘A Methodology of Unease’, in *Researching War: Feminist Methods, Ethics and Politics*, ed. Annick Wibben (London: Routledge, 2016), 117–40.

57. Lee Ann Fujii, *Interviewing in Social Science Research: A Relational Approach* (New York, NY: Routledge, 2018).

58. Julia Margaret Zulver, ‘Complex Gendered Agency in Mexico: How Women Negotiate Hierarchies of Fear to Search for the Disappeared’, *European Journal of Politics and Gender* 7 (2023): 1–17.

59. Julia Zulver et al., ‘Connecting, Venting, and Doing the “Behind the Scenes” Work: Bringing Feminist and Decolonial Insights to a Comparative Digital Data Collection Project’, *Qualitative & Multi-Method Research* 202 (2024), 41–53.

60. Marie E. Berry, ‘Radicalising Resilience: Mothering, Solidarity, and Interdependence among Women Survivors of War’, *Journal of International Relations and Development* 25 (2022): 946–66.

61. Cynthia Enloe, ‘Afterword: Being Reflexively Feminist Shouldn’t Be Easy’, in *Researching War: Feminist Methods, Ethics and Politics*, ed. Annick Wibben (London: Routledge, 2016), 258.

62. Judith Stacey, ‘Can There Be a Feminist Ethnography?’, in *Women’s Words: The Feminist Practice of Oral History*, eds. Sherna Berger Gluck and Daphne Patai (New York, NY: Routledge, 1991), 115.

63. Tapias Torrado, ‘Indigenous Women Leading the Defence of Human Rights from the Abuses by Mega-Projects in Latin America, in the Face of Extreme Violence’.

64. Ibid.

65. United Nations General Assembly, ‘United Nations Declaration on the Rights of Indigenous Peoples’, 2007. Available at: http://www.un.org/esa/socdev/unpfii/documents/DRIPS_en.pdf.

66. Government of Canada, ‘Tri-Council Policy Statement: Ethical Conduct for Research Involving Humans – TCPS 2 (2022) – Chapter 9: Research Involving the First Nations, Inuit, and Métis Peoples of Canada’, 2022. Avaialble at: https://ethics.gc.ca/eng/tcps2-eptc2_2022_chapter9-chapitre9.html#b.

67. Anctil Avoine, ‘Insurgent Peace Research: Affects, Friendship and Feminism as Methods’.

68. Marie E. Berry, *War, Women, and Power: From Violence to Mobilization in Rwanda and Bosnia-Herzegovina* (Cambridge: Cambridge University Press, 2018).

69. We thank Reviewer 1 for giving us the language with which to express these sentiments.

70. McKittrick, *Dear Science and Other Stories*.

71. Ibid., 36.

72. Ahmed, *Living a Feminist Lift*.

73. Blaney and Tickner, ‘Worlding, Ontolgical Politics, and the Possibility of a Decolonial IR’, 303.

74. Tamale, *Decolonization and Afro-Feminism*, 63.

